# An Overview of Nanoemulgels for Bioavailability Enhancement in Inflammatory Conditions via Topical Delivery

**DOI:** 10.3390/pharmaceutics15041187

**Published:** 2023-04-07

**Authors:** Diwya Kumar Lal, Bhavna Kumar, Abdulaziz S. Saeedan, Mohd Nazam Ansari

**Affiliations:** 1 Faculty of Pharmacy, DIT University, Dehradun 248009, Uttarakhand, India; 2 Department of Pharmacology and Toxicology, College of Pharmacy, Prince Sattam Bin Abdulaziz University, Alkharj 11942, Saudi Arabia

**Keywords:** anti-inflammatory, nanoemulsion, nanoemulgel, pharmacokinetics, pharmacodynamics

## Abstract

The anti-inflammatory drugs that are generally available possess the disadvantage of hydrophobicity, which leads to poor permeability and erratic bioavailability. Nanoemulgels (NEGs) are novel drug delivery systems that aim to improve the solubility and permeability of drugs across the biological membrane. The nano-sized droplets in the nanoemulsion enhance the permeation of the formulation, along with surfactants and co-surfactants that act as permeation enhancers and can further improve permeability. The hydrogel component of NEG helps to increase the viscosity and spreadability of the formulation, making it ideal for topical application. Moreover, oils that have anti-inflammatory properties, such as eucalyptus oil, emu oil and clove oil, are used as oil phases in the preparation of the nanoemulsion, which shows a synergistic effect with active moiety and enhances its overall therapeutic profile. This leads to the creation of hydrophobic drugs that possess enhanced pharmacokinetic and pharmacodynamic properties, and simultaneously avoid systemic side effects in individuals with external inflammatory disorders. The nanoemulsion’s effective spreadability, ease of application, non-invasive administration, and subsequent ability to achieve patient compliance make it more suitable for topical application in the combat of many inflammatory disorders, such as dermatitis, psoriasis, rheumatoid arthritis, osteoarthritis and so on. Although the large-scale practical application of NEG is limited due to problems regarding its scalability and thermodynamic instability, which arise from the use of high-energy approaches during the production of the nanoemulsion, these can be resolved by the advancement of an alternative nanoemulsification technique. Considering the potential advantages and long-term benefits of NEGs, the authors of this paper have compiled a review that elaborates the potential significance of utilizing nanoemulgels in a topical delivery system for anti-inflammatory drugs.

## 1. Introduction

Inflammation is a defensive measure against external stimuli or internal cellular damage that causes the liberation of immune response mediators at the site of injury. Normal inflammatory defense mechanisms have the potential to fight invaders, such as germs, foreign objects, or cancer cells, by attacking and degrading them. It is advantageous to get rid of the unwanted substance and start the tissue’s healing. A devastating level of inflammation can harm healthy tissues and result in chronic inflammation, which is associated with the etiology of a wide range of inflammatory disorders of the skin, such as atopic dermatitis, contact dermatitis, eczema, psoriasis, rosacea and seborrheic dermatitis [1,2,3,4]. Apart from inflammatory diseases of the skin, other major inflammatory disease conditions include rheumatoid arthritis (RA) and osteoarthritis [5,6]. Inflammatory disorders can have a detrimental effect on patients’ quality of life and can have a substantial socioeconomic impact [7]. The primary mediators of the inflammatory response include a number of cytokines, namely interleukin (IL)-1α, IL-1β, IL-6, IL-8, and tumor necrosis factor alpha (TNFα). Therefore, compounds that have the capacity to reduce excessive pro-inflammatory cytokine production or halt the inflammatory cascade have the potential to be used in the therapeutic treatment of skin inflammation [8].

Frequently employed drugs for the treatment of inflammation include non-steroidal anti-inflammatory drugs (NSAIDs), such as ibuprofen [9], ketoprofen, (S)-naproxen [10], nimesulide [11], meloxicam and celecoxib [12], some plant-derived polyphenols [13], such as quercetin [14], curcumin [15] and chrysophanol [16], and some polyphenol-derived alkaloids, such as capsaicin [17] and piperine [18]. More than 50% of the synthetic and herbal agents/compounds derived with the aim of treating both acute and chronic inflammation possess poor aqueous solubility, which restricts their applicative use. Due to solubility issues, the development of a dosage form remains a major challenge. Poor water solubility also leads to low bioavailability, which demonstrates the low therapeutic action of these compounds despite their extraordinary potential [12,19,20,21,22,23]. The low oral bioavailability of recently developed drug molecules may lead to fluctuations in both their intra- and inter-subject pharmacokinetics, and lead to poor dose constancy and irregular absorption [21,24].

Numerous methods have been developed to resolve the issues of low solubility and bioavailability. For instance, many delivery systems have been developed, pharmacologically active molecules have been chemically or physically altered, citrus pectin has been encapsulated into a micro/nano delivery system, and research has been conducted on salt formation, solid dispersion, size reduction, crystal engineering and complexation, and so on [25,26,27]. Amongst them, lipid-based formulations have drawn much attention for their ability to enhance the solubility of lipophilic drugs. This involves the development of formulations using carrier systems such as liposomes, niosomes, solid–lipid nanoparticles, macroemulsions, nanoemulsions, etc. Liposomes are vesicular carriers in which the active moiety is loaded inside a bilayer comprised of phospholipid and cholesterol. However, liposomes have shown issues regarding their stability in the alimentary canal and have shown low permeability throughout the intestinal epithelia [28,29]. Niosomes are vesicles that are made up of non-ionic surfactants, have issues regarding their physical stability, have a high risk of vesicle agglomeration, drug leakage, or hydrolyzing the encapsulated drugs, and are expensive to yield [30,31,32]. The major concerns surrounding lipid nanoparticles such as SLNs and NLCs are their low drug-loading capacities, lack of robust controlled drug release mechanisms, and restricted ability to perform transdermal drug delivery [33].

Comparatively, emulsion-based formulations are best suited to addressing issues regarding poor solubility and bioavailability [34,35]. The use of nanoemulsions (NEs), which are currently applied in established drug delivery systems, is a promising alternative delivery strategy for lipophilic drugs and could improve both their permeability and bioavailability by enhancing topical absorption [36]. Nanoemulsions possess various advantages that increase their ability to enhance skin permeation, such as nanosized droplets and lowered interfacial tension. Moreover, the oils used in the NE formulation not only aid in solubilizing medicines that are inadequately water-soluble, but also have the potential to increase skin permeability [37,38]. Despite all of these advantages, the utilization of NEs is restricted because of their low viscosity, spreadability, and skin retention [39].

This review summarizes the benefits of developing nanoemulgels that can be used in a delivery system designed specifically for anti-inflammatory drugs of both synthetic and herbal origin that are lipophilic or have poor water solubility, via the topical route. It includes a concise description of the building blocks of the nanoemulgels, along with the criteria for their selection and the steps involved in their preparation. Recent research studies on nanoemulgels have been compiled, showcasing the significant improvements made regarding permeation and bioavailability in the treatment of inflammatory conditions, including arthritis, dermatitis and psoriasis. Lastly, the current status of nanoemulgels regarding their ability to reduce inflammation, along with their future prospects, are discussed.

## 2. Nanoemulgel, a Potential Carrier System

Nanoemulgels (NEGs) are composed of nanosized emulsions (either oil/water or water/oil) that are integrated into a gel-based system. NEGs convert nanoemulsions into a more stable, non-greasy, and thicker system using a gelling agent, utilized in the preparation of the gel base [39]. Individually, both the components have their shortcomings; for instance, NEs have low viscosity and spreadability, inadequate skin retention and are non-scalable [40,41], while gels are not able to incorporate hydrophobic molecule effectively [42]. The drawbacks of both these systems can be resolved using a novel approach involving NEGs. With these, the lipid-soluble drugs are solubilized in the nanoemulsion’s oil phase, which is then combined with the gel to formulate an NEG. This allows the lipophilic drug to be incorporated into a hydrogel, while increasing the nanoemulsion’s viscosity at the same time [27]. The nanoemulsion component of the NEG safeguards the drug from hydrolysis and enzymatic degradation. Meanwhile, the gel component stabilizes the system’s thermodynamics by lowering the surface and interfacial tension, and increasing the viscosity and spreadability [43]. Lipophilic molecules are easily formulated into NEs, which can increase the permeability of drugs so that they can cross the layers of skin; this is because the distribution of nanoemulsion droplets covers a large surface area on the skin. As a result, remarkable enhancements have been made to the pharmacokinetic and pharmacodynamic aspects of lipophilic drugs [22]. Drawbacks related to traditional topical formulations, such as powders that show hygroscopicity, creams that show instabilities, such as phase inversion or breaking, in their formulations, components in ointments that go rancid, lotions that are sticky in nature, etc., lead to patient incompliance. There are no such drawbacks observed with NEG formulations [22,44].

In recent years, the development of nanoemulgels has emerged due to their numerous advantages; for example, they allow site specificity, continuous delivery, the release of drugs via a two-step mechanism, first from the nanoemulsion and then from the gel, and a reduction in the dose and dosing frequency. As a result, the total effectiveness of drug administration via the topical route is improved. NEGs do not suffer from drug-leaching or degradation problems regarding niosomes and liposomes, which also leads to their greater drug-loading efficiency. NEGs have the ability to control the release of a drug for an extended period of time, which is desirable for compounds with a short half-life. They create a greater concentration gradient and improve skin permeation due to their superior capacity to adhere to the skin and their greater drug solubilizing ability. Moreover, their noninvasive administration, bypassing GI side effects, makes their application easy and awards them excellent safety and therapeutic profiles, which ultimately leads to better patient compliance [22].

NEGs, used as a carrier system, are employed for the treatment of a number of inflammatory skin conditions, including acne fungal infections, pimples and psoriasis, along with osteoarthritis and rheumatoid arthritis-related inflammation. The literature reports that, as well as their topical use, nanoemulgels can be used for local and systemic diseases/disorders such as alopecia, periodontitis, and Parkinson’s if administered via ocular, dental, vaginal, and nose-to-brain routes [21].

## 3. Nanoemulgels in Topical Delivery for Anti-Inflammatory Drugs

**Figure 1:**
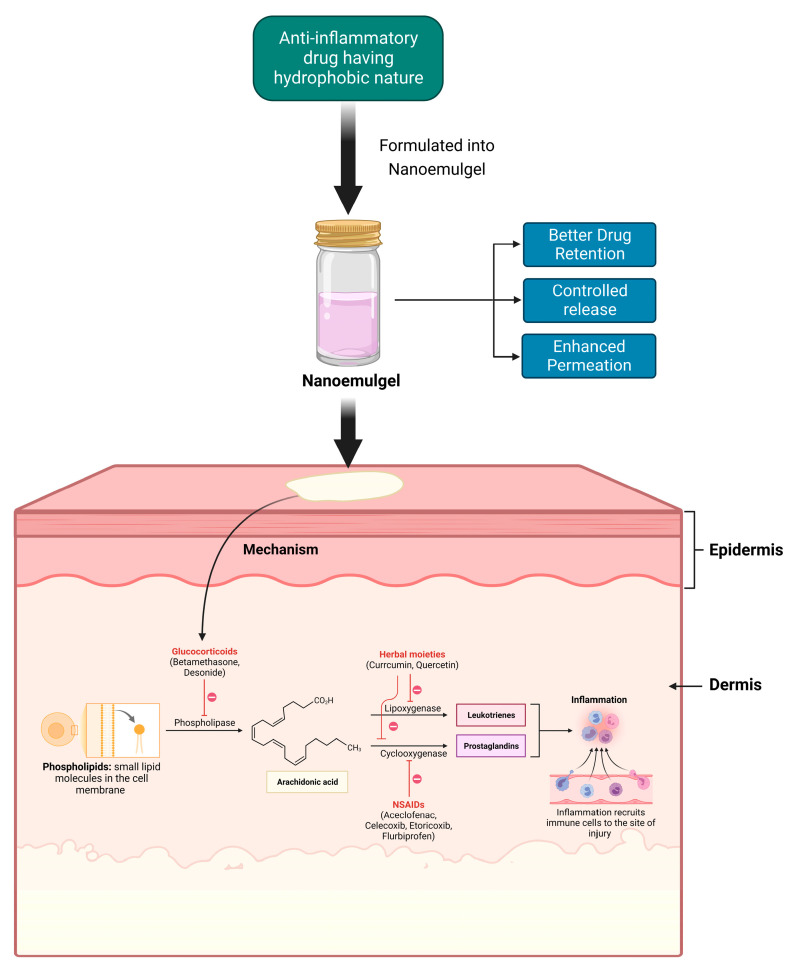
Delivery of anti-inflammatory drug along with its mechanism of action.

Anti-inflammatory drugs showcase their activity at different stages of the inflammatory cascade (Figure 1). Glucocorticoids inhibit the phospholipase enzyme that catalyzes the formation of Arachidonic acid, which is the precursor of inflammatory mediators such as prostaglandins and leukotrienes [45,46]. NSAIDs primarily inhibit cyclooxygenase 1 (COX1) and cyclooxygenase 2 (COX2), which, in turn, restrict the formation of prostaglandins, which are responsible for inflammatory reactions [47]. Meanwhile, many natural anti-inflammatory moieties such as curcumin and quercetin have been reported to have both COX and lipoxygenase (LOX)-inhibiting properties [14,48]. Topical delivery is an excellent approach for the management of external inflammatory conditions, because by opting for this route, we can not only bypass the first-pass metabolism, but also the gastrointestinal (GI) barriers; these are the gastric emptying time and the intestinal transit time, the enzymes present and pH changes [49,50]. Topical NSAID application has the benefit of improving the distribution of local drugs to the damaged tissues, while minimizing the risk of systemic side effects, such GI bleeding and peptic ulcers [51]. Additionally, oils that possess anti-inflammatory properties can be meticulously chosen as the oil phase for the formulation; this can synergistically enhance the desired outcome of the formulation.

The nanosized globules of the chosen NE enhance the potency of the NEG by improving its permeability and diffusibility through the use of appropriate permeation enhancers [43]. Moreover, a high concentration of water in the gel can sufficiently hydrate the stratum corneum (SC) and cause its tight architecture to leak, allowing the active ingredients to readily penetrate the skin [52]. Before reaching the skin’s surface, the drug in the NE first traverses from the internal phase (Nanoemulsion) to the external phase (gel), and thus serves as a drug reservoir for topical delivery. When applied topically, oily globules first get liberated from the hydrogel, then permeate deep into the SC of the skin, where they deliver the drug moiety [23].

### Mechanistic Approach of Nanoemulgel Delivery via Topical Route

**Figure 2:**
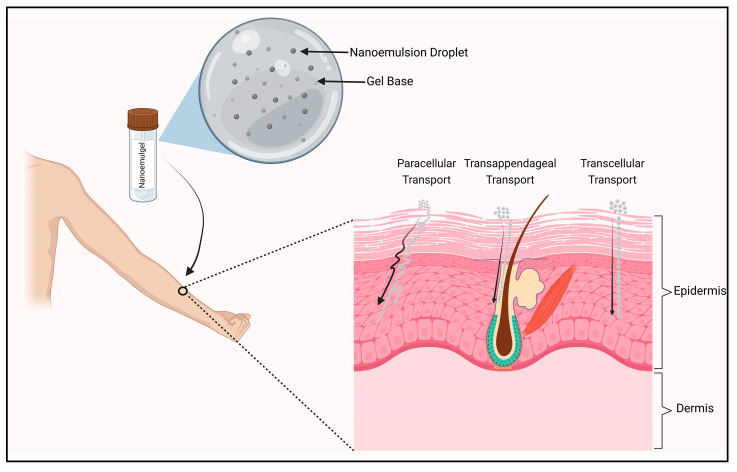
Mechanistic representation of nanoemulgel delivery via skin.

The stratum corneum is regarded as the most effective barrier for regulating the entry and permeation of topically applied drugs. Tight junctions act as a secondary barrier when the subcutaneous (SC) layer is disturbed due to skin diseases or permeation enhancers [53,54]. The permeation of the drug molecule takes place via three routes, namely. paracellular, transcellular and transappendageal routes (Figure 2). The primary pathway for permeation through skin is the paracellular route, which involves the drug traversing via the lipid milieu in among the corneocytes. To pass through this pathway, small lipophilic molecules (molecular mass < 500 Dalton) can penetrate the tight lipid junctions between the cells in a circuitous manner [55]. The transcellular pathway enables the direct passage of the molecule through the SC into the inner layers of the epidermis and possibly to the dermis at the bottom. A molecule moving through the transcellular pathway needs to partition into and diffuse via corneocytes for up to 20 lipid lamellae, which separate each of these cells in order to pass across corneocytes. Thus, to pass through the transcellular pathway, the drug or the carrier should have both hydrophilic and hydrophobic properties. The transcellular route inhibits the permeability of highly lipophilic molecules while allowing tiny hydrophilic or modestly lipophilic compounds (log p between 1–3) to permeate across the epidermis [55,56]. The transappendageal route involves transporting compounds along hair follicles and their accompanying sebaceous glands, as well as sweat glands. It is comprehensively understood that the appendages’ (hair follicles and associated glands) contribution to epidermal permeation is often minimal because they only make up a small portion of the skin (e.g., only around 0.1% of the forearm skin) for permeation [57]. NEGs enhance the permeation of the drug as it can utilize all three pathways to transport the drug through the epidermis. The formulation consists of oils, surfactants alone or in combination with the cosurfactant, which acts as an inherent permeation enhancer and gelling agent which aids in increasing the permeability by enhancing the formulation’s adhesion to the skin.

Various researchers have conducted extensive research in order to assess the ability of a plethora of drugs to permeate the skin using NEGs. Alterations in the epidermis, such as the growth of plaque or scaling, which serve as barriers to drug penetration across skin, generally result in decreased drug permeation through the psoriatic skin [58]. According to Somagoni et al., NEG has the capacity to transport drugs across skin that is already inflamed with psoriasis in order to effectively inhibit inflammation, as their formulation containing aceclofenac and capsaicin demonstrated permeation that was almost twice as good their marketed product across the dermatomed skin [59]. Azeem et al. reported penetration of the skin that was 7.5 times better than that of a regular hydrogel made from a ropinirole-loaded NEG. Additionally, ropinirole had an extended release, and it had twice the bioavailability of the marketed gel formulation [60]. Hussain et al. formulated an amphotericin-B-loaded NEG that achieved the maximum percutaneous penetration flux compared to the nanoemulsion and drug solution; this NEG also showed prolonged drug release, as demonstrated by in vitro drug release [61]. In another study by Begur et al., a tacrolimus-loaded NEG demonstrated considerably superior drug permeation and flux, alongside a reduced lag time and skin drug retention, compared to the marketed preparation [62]. Wais et al., reported an almost fourfold increase in bioavailability when a glibenclamide-loaded nanoemulsion was applied topically, compared to an oral suspension of same drug. The formulation exhibited high T_max_ and C_max_ values with a steady plasma drug concentration, as revealed by a pharmacokinetic study [63]. Similarly, Singh et al. were able to increase the area under the curve by 1.72-fold, as examined by the pharmacokinetic study of Carvedilol-loaded NEG, compared to the conventional oral tablet [64]. All of this research establishes the ability of NEG formulations to enhance permeation and bioavailability.

## 4. Components of Nanoemulgel

**Figure 3:**
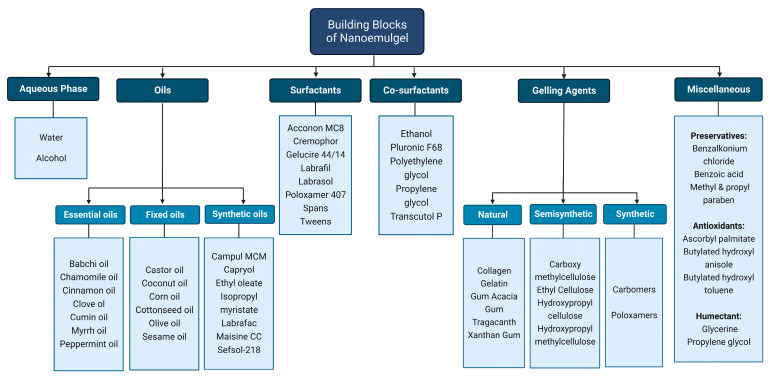
Concept diagram for the building blocks required to design an NEG.

Nanoemulgels are basically a two-component system, i.e., NEs are incorporated into the gel base. Nanoemulsions can be a nanosized emulsion of the o/w or w/o type, made up of a blend of oil and water with the help of a suitable surfactant and co-surfactant. The gel base simply consists of polymers with the ability to swell when they encounter liquid by absorption [39,65]. An overview of the essential components of an NEG is given below (Figure 3):

### 4.1. Aqueous Solvents

Aqueous solvents constitute the aqueous phase of the nanoemulsion and the major portion of the gel part of the NEG. The most common aqueous solvents in use are water and alcohol [22].

### 4.2. Oil Phase

They serve as a medium for dissolving or dispersing numerous constituents of the formulation. Mostly, lipids of either natural or synthetic origin are used as the oil phase in medicinal and cosmetic applications, except if the oil itself is an active agent. Lipids can range in consistency from liquids to high-molecular-weight solids. A stable emulsion is formed when an oil has a high level of hydrophobicity and shows better emulsification properties, and can simultaneously reduce the solubility of the lipophilic molecules. Therefore, selecting an oil is a crucial preliminary step in the production of an NEG as a novel nano-drug delivery system. The suitable oil should be chosen depending on the formulation specifications for permeability, solubility, stability, and viscosity. Generally, oils in which drugs are more soluble and having good permeation-enhancing properties must be selected. Mineral oils are typically employed for the administration of lipophilic drugs in topical emulsions due to their occlusive properties, which help to stop transepidermal water loss (TEWL) from the skin [66,67].

The oil selection may be affected by the additional medicinal effects that the oil can bring to the formulation. Certain oils of natural origin have proven anti-inflammatory properties, which can show synergistic activity with the active pharmaceutical ingredient and thus enhance the overall anti-inflammatory efficacy of the formulation. Sometimes the oil itself is potent enough to impart the therapeutic action that no other active moiety can add to the formulation. For example, Eid et al. prepared an NEG using Swietenia macrophylla oil (anti-inflammatory oil) as the oily phase in the preparation of the nanoemulsion [68]. Hence, the oil can be chosen critically to compliment the pharmacological activity of the formulation.

### 4.3. Surfactants

Surfactants that are helpful in the emulsification process, thus named emulsifiers, can be added to stabilize the oil and water phases together [69]. They work at the interface by lowering the tension between the two phases, which is important for the development of dispersion, and they also aid in reaching the distribution equilibrium at the interface, resulting in the increased thermodynamic and kinetic stability of the emulsion [21,70]. As a result, they have good solubility in both water and oil. “The portion in which the surfactant is more soluble produces the external phase of the emulsion”, states the Bancroft rule [71]. For instance, sorbitan fatty acid esters that are oil-soluble favor the production of w/o emulsion, while tweens that are water-soluble facilitate the production of the o/w type of emulsion [39,72]. In addition, surfactants possess permeation-enhancing properties. An ionic surface-active agent interacts with the keratin protein of the epidermis, loosens the tight keratin framework and enhances the permeation. A nonionic surface-active agent can enter the intercellular areas of the SC, enhance its fluidity, and ultimately dissolve and remove lipid constituents [73]. The suitable surfactant for an NEG is chosen depending on its stability, biocompatibility, high drug-loading capacity, and effective emulsification capabilities. When choosing a surfactant, toxicity issues must be kept in mind because, depending on the administration route, it may cause irritation to the skin or gastrointestinal system. In general, ionic surfactants are not recommended for use because of their toxicity and lack of biocompatibility [70,74]. The most commonly used surface active agents include Tween 80, Tween 20, Poloxamer 407, etc.

### 4.4. Cosurfactants

Cosurfactants are added to assist surfactants when obtaining nanoemulsion systems at low surfactant concentrations. The cosurfactant and surfactant can cooperate to aid in the emulsification process by disturbing the interfacial layer and facilitating the solubilization of oil [75]. The co-surfactant and the concentration to be used have to be decided meticulously because they may impact the surfactant’s ability to emulsify the system. Cosurfactants and surfactants whose values are close in the HLB scale cannot produce a stable emulsion in the same way that those produced by non-ionic surfactants with distinct HLB values can. This can be explained, as the higher HLB value surfactants may dissolve in the aqueous phase, while lower HLB value surfactants may dissolve in the non-aqueous phase, allowing for a more effective alliance between the surfactant and the cosurfactant mixture [76]. The most preferable co-surfactants are those based on alcohol because they can partition between both the aqueous and oil phases, enhancing the drug’s solubility. Carbitol, ethanol, propylene glycol, polyethylene glycol, and transcutol P are some of the most commonly employed cosurfactants in the formation of nanoemulsions [77].

### 4.5. Gelling Agents

Gelling agents are introduced into the formulation to transform the nanoemulsion formulation’s liquid state to gel and address the issues of low viscosity, limited spreadability, and poor retention [18]. A gelling ingredient, when dispersed in liquid (mostly aqueous phase), results in a weakly cohesive gelling matrix that stabilizes the formulation and promotes the optimum percutaneous drug delivery when utilized in an externally applied formulation [78]. The gelling agent’s thixotropic property thickens the formulation without changing its volume, aiding in the transition of the liquid to gel. Some gelling agents work by dissolving in the liquid phase and producing a colloidal mixture that may cross-polymerize to construct a minimally cohesive internal structure. Other gelling agents work by virtue of their thixotropic property, i.e., their time-dependent shear-thinning ability, which causes discrete particles to adhere or interlock to withstand strain. Gelling agents have a crucial role in determining the number of parameters, which can include bioadhesion, consistency, drug-release kinetics, extrudability, homogeneity, rheological properties, swelling index, and the spreadability of nanoemulgels. Sodium Carboxy methylcellulose (NaCMC), Carbomers and Hydroxypropyl methylcellulose (HPMC) are the most commonly used gelling agents. Plant-derived polysaccharides that act as stimuli-responsive gelling agents can be used for preparing a gel base that allows the delivery of drugs in a more targeted manner [79,80]. These kinds of polymers are able to interchange their phases, alter their rigidness, and control and sustain their drug delivery. They can be made to release the drug only if specific conditions (stimuli) are met; this includes in a ROS (Reactive Oxygen Species)-rich environment during inflammation and tissue injury [81], when there is a lower extracellular pH in inflamed tissue or around a tumorous microenvironment [82], or when temperature is increased, which acts as an indicator of the inflammatory phase in wound progression [80,83]. Hydrogel systems, such as ROS-scavenging hydrogels, that utilize polyvinyl alcohol attached to a ROS-responsive linker have been developed; these are able to suppress the inflammation and reduce the release of numerous pro-inflammatory cytokines [84].

### 4.6. Miscellaneous Additives

Miscellaneous additives are optional additives that can be used when designing the formulation of an NEG and are only used if the stability of the formulation under an adverse external environment needs to be enhanced. Preservatives are included in the preparation to protect the formulation against microbial degradation and prolong the stability of the preparation. Preservatives, including benzalkonium chloride, benzoic acid, methylparaben, phenoxyethanol, and propylparaben, are some of those most widely used. Antioxidants are employed to stop the oxidative destruction of the formulation and its components. It includes compounds such as ascorbyl palmitate, butylate hydroxyl anisole and butylate hydroxyl toluene. Humectants help to retain moisture by reducing the rate of dehydration. This protects the gel from drying out. Glycerin is the most common humectant in the topical formulation. [19,70,71].

## 5. Stages of Nanoemulgel Formulation Design

Firstly, preformulation studies for the screening of oils, surfactants and cosurfactants, gelling agents and the determination of their optimal amounts should be carried out. NEGs can be basically prepared in a two-step procedure; this involves the preparation of the nanoemulsion and its subsequent incorporation into the gel base for maintaining the rheological properties that are needed to achieve topical delivery, as described below in brief.

### 5.1. Preformulation Studies

The final composition of the formulation should be critically selected based on the results of the preformulation studies. In this step, the oily phase is selected based on its ability to dissolve the drug moiety. The selection of the ratios used for the surfactant and cosurfactants is based on the characteristic parameters used to develop the nano-sized emulsion and their compatibility with the oil and the type of emulsion (o/w or w/o). One technique used to critically analyze whether the concentration of these components is able to form a nanoemulsion involves plotting a pseudoternary phase diagram. This phase diagram represents the ratio of these three components, at which stable nanoemulsion has been developed as the nanoemulsification region [72]. This ratio is further utilized to formulate and optimize the nanoemulsion.

### 5.2. Preparation of Nanoemulsion

**Figure 4:**
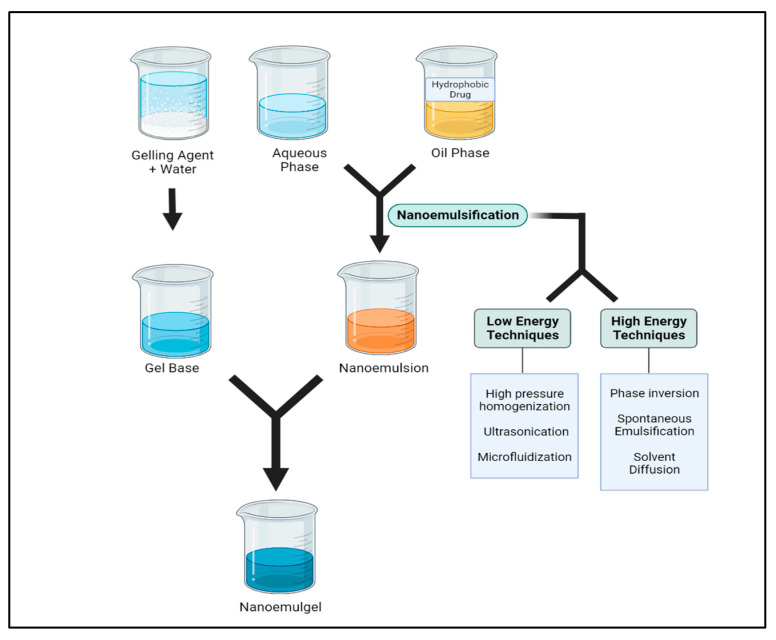
Steps to formulate the nanoemulgel.

The drug, surfactant and cosurfactant are dissolved based on their solubility either in the chosen oil phase or aqueous phase. The oil and aqueous phases are heated separately, and then they are combined by gradually adding one into the other while being constantly stirred until they reach room temperature, as shown in Figure 4.

The nanoemulsion may be formulated by either low- or high-energy methods. Low-energy approaches include self-nanoemulsification and phase inversion (phase inversion temperature (PIT), phase inversion composition (PIC)), and the emulsification and solvent diffusion technique; meanwhile, high-energy techniques involve ultrasonication, microfluidization and high-pressure homogenization. The use of low-energy procedures over high-energy ones is advised because they are more effective and don not necessitate the use of complex equipment [85].

The particle size can be controlled and regulated using a variety of formulation compositions by applying high-energy techniques. The stability, rheology, and color of the emulsion can also be controlled using high-energy techniques. [85]. High mechanical energy is employed to generate intense disruptive forces that split up big droplets into nano-sized droplets and create nanoemulsions that have a high kinetic energy [86]. The high-energy approach uses mechanical tools to generate an intensely disruptive force that makes both phases experience a size reduction. Therefore, this technique may result in the overheating of the formulation’s components, which would lead to the thermodynamic instability of the emulsion and make it unsuitable for drugs that are thermolabile [65].

Low-energy emulsification methods are characterized by their ability to leverage the internal chemical energy of the system, resulting in greater energy efficiency [87]. This approach involves less energy, preventing the degradation of heat-labile components [88]. The low-energy or spontaneous approach is frequently used to produce essential oil-based nanoemulsions and stop the volatile chemicals in essential oils from evaporating [89].

### 5.3. Preparation of Gel Base

The preparation of gelling media involves dissolving gelling agents into an aqueous medium until complete swelling is achieved. For this, the selected polymer is dispersed in pure water while being continuously stirred by mechanical means at specified condition for a specific time and a constant rate on order to achieve complete swelling. Lastly, the gel base is adjusted for pH, which can be delivered effectively to the topical system [40].

### 5.4. Incorporation of Nanoemulsion into Gel Base

Any of the aforementioned processes can be used to create the nanoemulsion, which can then be transformed into an NEG by employing a gel base. In order to incorporate a nanoemulsion into the gel matrix, the gel and nanoemulsion at a fixed ratio are gradually mixed and continuously stirred to maintain homogeneity. When a gelling component is introduced to an o/w nanoemulsion solution, it gets thickened to produce gel. This is because the agent’s thixotropic properties aid in the formulation’s transition from a gel to a solution when shear force is applied, without affecting its volume [40].

## 6. Evaluation Properties of Nanoemulgels

NEGs are semisolid formulations that comprise a nano-sized emulsion and gelling media, making them appropriate for topical application. A wide range of analysis methods are needed to characterize the different physicochemical features of NEGs. The evaluation of NEGs covers the analysis of both of their constituting systems, namely the nanoemulsion and gel. The nanoemulsion portion of the NEG needs to be analyzed for its droplet size, polydispersity index (PDI), and zeta potential (Table 1). Moreover, the final emulgel formulation needs to be evaluated for its pH, rheological properties, spreadability, bio-adhesion, skin irritation study, in vitro drug release, ex vivo permeation study and in vivo studies, which can be performed to fully comprehend the performance of the NEG. A brief description of the important parameters involved in the technique for examining the critical features of NEGs are discussed below and mentioned in Table 1 [65,90]:

### 6.1. Droplet Size and Poly Dispersity Index (PDI)

The droplet size of an NEG is known as its hydrodynamic diameter, which corresponds to the diameter of an analogous rigid sphere that diffuses at an equivalent rate to that of the drug molecule [91]. The PDI is used to determine the size distribution of the nanoemulsion droplet and can be calculated as the ratio of the standard deviation of the droplet size to the mean droplet size. The droplet size and PDI not only affect the drug release and formulation stability, but also the ex vivo and in vivo performance of the formulation. Furthermore, it is also critical to assess batch to batch consistency. If the nanoemulsion is between 50 and 200 nm in size, the solution is clear and transparent, and if it is 500 nm or larger, the solution appears milky or hazy. The dynamic light scattering (DLS) technique is specifically used for the measurement of globule size, while the zeta sizer or master sizer can be employed to measure both the size and PDI [92,93].

### 6.2. Zeta Potential

Nanoemulsions possess electrical charge because of the presence of several kinds of surfactants in their formulation. The magnitude of the repulsion increases with zeta potential, enhancing the formulation’s stability. For instance, emulsion globules are prevented from aggregating due to having a high zeta potential. The surface charge can be altered with a surface charge modifier. For instance, the zeta potential becomes positive if a positively charged modifier is utilized and vice versa [82]. Thus, surfactants (anionic or cationic) are crucial for emulsion stability. The zeta potential can be evaluated using a variety of devices, including the Malvern Nanosizer/Zetasizer^®^ nano-ZS ZEN 3600, the ZC-2000 and others [81].

### 6.3. pH

The pH simply describes the acidity or basicity of a formulation. In case of topical formulations, a pH that is too high or too low may cause adverse effects to the skin surface, such as irritation or allergy. It also affects the stability of the drug and its release from the formulation. Ideally, the pH of the formulation should be in accordance with that of the skin’s, i.e., ranging between 4–7. Digital pH meters can be employed for the measurement of pH [83].

### 6.4. Rheological Properties

Rheological studies are responsible for the analysis of the flow and deformation of materials in response to external stress or force. A rheological analysis of materials indicates how concentrations of different excipients, such as oils, surface-active agents, and gelling agents, affect the flow behavior of the formulation. Variations in the viscosity and flowability of the formulation may affect its stability, spreadability, drug release, and other in vivo factors. For instance, the shear-thinning properties of a formulation create a thin layer on the surface of the skin, thus enhancing permeability, whilst a viscous formulation reduces permeation. Because of this, the rheological properties are a critical parameter in the development of NEGs, and they may be assessed using a variety of viscometers, such as the Brookfield viscometer, or rheometers [90].

### 6.5. Spreadability

Spreadability is an evaluation of the uniform spreading of the NEG over the skin surface, ensuring the even distribution of the dosage form, and preventing stranded dose delivery that would otherwise impair efficacy. The spreadability is significantly impacted by the nanoemulgel’s viscosity. For assessing the spreadability, there is currently no accepted standard procedure. The parallel plate approach (also known as the slide and drag method) is a widely used technique because it is straightforward and can be modified according to the requirements. In this approach, the sample is placed in the marked center of a glass slab and another slab is placed parallel above it. A certain amount of weight is placed above it and is left alone for a specified time interval. The spreadability is calculated by the formula given below:

Spreadability (S) = M × L/T

where M is the weight attached to the upper glass slab, L is length of the glass slabs, and T corresponds to the time required to separate the slabs [94].

### 6.6. Adhesive Property

The amount of force necessary to separate the drug delivery system from a biological surface is calculated using the bio-adhesive strength. This characteristic is essential for a topical dose form if extended contact is desired. Rat or pig skin is typically used for this test due to its similarity to human skin. Although there are numerous methods to evaluate adhesive strength, none of them have FDA approval. One such approach uses a texture analyzer, which uses an upper mobile and a static bottom base plate, both covered by skin. The base plate’s skin is covered with the formulation. The upper plate is slid down until it makes contact with the lower base plate and is held it in place for a minute at minimum. The upper probe is steadily elevated until the skin sheets separate. The equipment measures the force needed to separate the two skin sheets, and it displays that force as the area under the force–distance curve. It is quantified as the percentage increase in force required to separate the sheets with and without formulation [95]. The formula for the calculation is given below:

Percentage increase (%) = F _(Formulation−No Formulation)_/F _(No_ _Formulation)_ × 100

where F stands for the peak force required to separate the skin sheets.

### 6.7. In Vitro Drug Release

The in vitro performance of a formulation in terms of its drug release and dissolution is correlated to its therapeutic bioavailability, potency and safety. The in vitro dissolution test assesses the drug release from nanoemulgels in a controlled environment that mimics the body (e.g., kind of membrane, temperature, volume and pH of dissolving media, agitation rate) in a time-dependent manner. The in vitro release test (IVRT) has the potential to demonstrate the combined impacts of a number of physicochemical properties, including globule size, viscosity, etc. [96]. The IVRT assembly comprises donor and receptor chambers, with a receptor membrane standing between them. The sample is kept in the donor chamber, while the receptor media are kept in the receptor chamber. A buffer or hydro-alcoholic solution can be used as the receptor media, depending on the drug’s solubility, sink condition, and stability. The membrane is chosen depending on its pore size, permeability, and anticipated inertness towards the drug molecule. The receptor membrane should, if required, be saturated with dissolution medium. The test temperature and pH are maintained at 32 ± 1 °C and 5.5, respectively, in the context of topical and transdermal formulations. The dissolution media in the receptor compartment is stirred with a Teflon-coated magnetic stirrer. The drug from the formulation is allowed to cross to the receptor compartment after being deposited in the donor section. The amount of drug entering the receptor compartment is calculated at a specified time interval. These data are plotted on a graph as a function of time, which displays the amount of drug released from the formulation at that particular time and the cumulative drug release from the formulation [97,98]. This is particularly important in order to assess whether the formulation shows an immediate release or sustained release. The data can further be modeled to reveal the formulation’s pattern of drug release, for instance, if it follows zero-order kinetics, first-order kinetics or some specific kinetics, such as the Higuchi or Korsmeyer–Peppas models.

**Table 1:** Key evaluation parameters for the nanoemulgel.

S. No.	Formulation	Characterization of Nanoemulsion			Characterization of Nanoemulgel			Reference
		Droplet Size (nm)	PDI	Zeta Potential (mV)	pH	Viscosity	Spreadability	
1	Minocycline-loaded NEG	90–201	0.348–0.563	−17.40 mV	5.40	-	5 cm/s	[99]
2	Atorvastatin-loaded NEG	148	0.3	-	-	85,900 ± 2050 cps	51 ± 0.66 mm	[100]
3	Thymoquinone-loaded topical NEG	40.02–99.66	0.052–0.542	−26.7–−30.6	5.53 ± 0.04	88.82–71.04 mPas	-	[101]
4	Coriandrum sativum oil NEG	165.72–189.59	0.177–0.243	Less than −35	6	-	-	[102]
5	Glimepiride-loaded NEG (recheck)	143.1–233.3	0.315–0.421	−12.8–−17.1	5.06 to 5.45	34.7–58.7 mPas	1.35–1.41 cm^2^/g	[103]
6	Quercetin-loaded NEG	173.1 ± 1.2	0.353 ± 0.13	−36.1 ± 5.99	5.8	100,803 ± 1234 cps	-	[104]
7	Tamoxifen citrate-containing topical NEG	29.65–95.73	8.1–16.3	0.163–0.163	5.55–5.57	-	1.27–1.29 cm^2^/g	[105]
8	Retinyl palmitate NEG	16.71–71.95	0.015–0.606	−19.03 to −20.36	5.53 ± 0.06	77.48–89.22mPas	1.34 ± 0.03 cm^2^/g	[106]
9	Fusidic acid-incorporated NEG	113.6 ± 3.21	-	-	6.61	25,265 cps	33.6 mm	[107]
10	Curcumin NEG	49.61–84.23	0.05–0.23	−15.96 to −20.26	-	83.74–89.82 mPas	-	[108]

## 7. Permeability and Bioavailability Enhancement of Anti-Inflammatory Drugs by Topical Nanoemulgel System

### 7.1. Herbal Anti-Inflammatory Compounds

In recent years, the use of herbal compounds has increased globally owing to their potential health benefits and lack of side effects compared to mainstream synthetic drugs. Despite the number of remarkable studies that report on in vitro pharmacological results regarding these compounds’ antioxidant, anti-inflammatory, anti-diabetic effects, there are several herbal compounds and extracts that exhibit minimal in vivo activity because of issues related to their solubility with lipophilic compounds or their inappropriate molecular size, which causes inadequate absorption and therefore poor bioavailability [109]. Nanoemulgels can overcome these shortcomings of herbal anti-inflammatory compounds and can fully unlock their potential, leading to an increased pharmacological response and minimal unwanted side effects [110]. A number of herbal anti-inflammatory compounds are being formulated into nanoemulgels to improve permeability and bioavailability, ultimately enhancing therapeutic efficacy. Detailed descriptions of these are provided below in Table 2.

### 7.2. Synthetic Anti-Inflammatory Compounds

Traditional anti-inflammatory therapy, including NSAIDs, steroidal drugs, etc., when administered systemically, has many drawbacks, including poor bioavailability, breakdown by gastric enzymes, the first pass effect, interactions with food, and toxicity. The localized and targeted administration of anti-inflammatory drugs is an appealing alternative because of their capacity to prevent off-target toxicities. However, in topical delivery, the skin serves as an effective barricade to the majority of polar molecule candidates, and epidermal absorption is typically too slow and insufficient to result in a clinically significant therapeutic benefit. Hence, there is a need to overcome this barrier in order to achieve effective bioavailability and ultimately the required therapeutic benefits. A novel formulation approach, such as that used with NEGs, can be employed to address the limitations that come in the way of the topical delivery of these drugs; indeed, the components used in their formulation can effectively help to enhance permeability through the epidermis [121,122,123]. Many researchers have formulated nanoemulgels that incorporate a variety of synthetic anti-inflammatory components, as described below in Table 3.

## 8. Toxicity Concerns

Nanoemulgels contain components such as surfactants and cosurfactants which, are known to cause skin irritation and deterioration, cytotoxicity, and allergic reactions in some patients. Anionic surfactants, such as sodium dodecyl sulfate (SDS), primarily target the SC layer of skin, causing damage to the bilipid layer, the denaturation of protein and skin dehydration, which may lead to dermatitis in sensitive skin. Cationic surfactants cause more severe cytotoxic effects and apoptosis in normal and cancerous cells. Conversely, non-ionic surfactants are relatively less toxic and irritative to skin [140,141]. Hence, non-ionic surfactants are preferred over ionic surfactants for topical formulations. Cosurfactants such as propylene glycol, when used in an unreasonably high amount, can cause hyperosmolarity, cardiac arrhythmia, lactic acidosis and toxicity of the central nervous system. Fligner et al. reported cardiorespiratory arrest due to a high propylene glycol concentration (1059 mg/dL) in an 8-year-old child during topical silver sulfadiazine therapy [142]. To minimize these types of toxic effects, these kinds of components should be used in a minimum quantity. When used in the proper amount, numerous researchers have reported that the nanoemulgels did not cause skin irritation or toxicity. Abdallah et al. conducted a skin irritation study by spreading a brucine-loaded nanoemulgel over the shaved skin of rat and covered it with gauze. The rats did not show any signs of oedema or erythema, even after 24 h of application. Similarly, Bhattacharya and Prajapati conducted an acute skin irritation study of a Celecoxib-loaded nanoemulgel on rabbits and reported a 1.6 times lower skin irritation score compared to that of a standard irritant after 7 days of application.

Moreover, the risk of experiencing systemic side effects caused by topically applied nanoemulgels cannot be ignored due to the possibility of the formulation reaching the blood stream by virtue of its enhanced permeation and nano size range, although extensive research needs to be conducted to reach conclusive findings regarding nanoemulgel toxicity. Nano formulations such as polymeric nanoparticles are associated with oxidative stress and cytotoxicity, lipid-based nanoparticles have shown hypersensitivity and cardiopulmonary distress, while tiny rare-earth fluoride nanoparticles can encourage tumor growth via electrical dipole interactions, which are inversely proportion to the size [143,144].

## 9. Current and Future Perspectives of Anti-Inflammatory Topical Nanoemulgel

Anti-inflammatory drugs with hydrophobic properties make it challenging to incorporate them into conventional dosage forms such as tablets, capsules, and syrups. These drugs tend to clump together and do not dissolve evenly in water, leading to inconsistent dosing and reduced efficacy. However, novel drug delivery approaches such as NEG have helped overcome this limitation. Extensive studies have been conducted on nanoemulgels, which show great potential in delivering synthetic and herbal anti-inflammatory compounds through topical means. Better thermodynamic and kinetic stability, improved permeability, and controlled release are a few of the remarkable features that make NEGs an attractive formulation for the delivery of such drugs. By critically choosing an oil phase that has inherent anti-inflammatory properties, many researchers have reported the enhanced synergistic effect and improved pharmacological profile of the NEG formulation compared to marketed products. Nanoemulgels have eliminated the use of excipients such as penetration enhancers and stabilizers owing to their nano size and the permeation-enhancing properties of its formulation components. Additionally, their qualities, such as effective spreadability, non-invasive administration, ease of application, and subsequent patient compliance, make it a more suitable option for topical delivery.

While NEGs have shown promising results, their practical application on a commercial scale is hindered by the challenge of mass production. Current methods of formulating NEGs on an industrial scale rely on high-energy approaches for the production of NEs, which can cause thermodynamic instability, especially for thermolabile drugs. Therefore, the development of more efficient and cost-effective techniques for producing stable NEs could greatly enhance their applicability for pharmaceutical use. The energy-intensive production of NEGs may be seen as a short-term obstacle, considering the potential advantages and the long-term benefits that this technology can offer. With fewer steps required for formulation, NEGs have the potential to be a more cost-effective and time-efficient option compared to other formulations. Very limited nanoemulgel patents for inflammatory conditions via topical applications have been reported, as mentioned in Table 4. Hence, this approach can be scaled up in order to market the product as a novel delivery system that can target inflammatory conditions via the topical route.

## 10. Conclusions

This review confirms that NEGs hold great promise as a novel drug delivery system for improving the percutaneous permeability and bioavailability of anti-inflammatory agents. Most anti-inflammatory medicines have a low aqueous solubility that causes unpredictable absorption, low oral bioavailability, and pharmacokinetic fluctuations. NEGs have demonstrated remarkable benefits for such medicines when compared to alternative formulations, especially in the instance of topical delivery. Due to their dual characteristics, i.e., the combination of a nanoscale emulsion and a gel base as a single formulation, nanoemulgels are best suited to the topical delivery of such drugs. Nanoemulsions can be developed by applying external energy to the heterogeneous mixture (high-energy emulsification) or by mixing the compositions by lowering the interfacial tension at the oil/water interface (low-energy emulsification). Further, via the addition of a wide variety of gelling agents, NEGs can be created from a nanoemulsion. The active moiety of the NEG is protected by the nanoemulsion system by the blocking of enzymatic degradation and certain processes, such as hydrolysis. By reducing the interfacial and surface tension, and increasing the aqueous phase’s viscosity, the gel base gives the emulsion thermodynamic stability. Due to the presence of globules in nano form, together with the use of certain penetration enhancers, the formulation’s effectiveness can be increased by improving diffusibility and permeability through the epidermal layer, thus enhancing the topical delivery of drugs. Moreover, it can bypass first-pass metabolism, target the site of action more precisely, and avoid gastric/systemic incompatibilities. Overall, the rheological properties of NEG’s, along with their enhanced patient acceptance, make them a promising system for various topical aliments. NEGs have been employed for the treatment of various topical/epidermal and dental inflammatory conditions, including psoriasis, rheumatoid arthritis, periodontitis, acne, dermatitis, etc. Despite having a few challenges ahead of it regarding long-term stability, mass production and commercialization, NEGs possess immense potential for the topical application of anti-inflammatory drugs.

## Figures and Tables

**Table 2:** Herbal drug-loaded nanogels for anti-inflammatory action.

S. No.	Active Moiety	Oil	Surfactant and Co-Surfactant	Gelling Agent	Indication	Inference	Reference
1	Brucine	Myrrh oil	Tween 80 and PEG 400	Carboxymethyl-cellulose sodium	Anti-nociceptive and Anti-inflammatory	NEG showed a higher cumulative drug release, enhanced transdermal flux and permeation, and thus enhanced invivo activity compared to conventional gels and emulgels	[111]
2	Chamomile oil	Chamomile and olive oil	Gelucire 44/14, Tween 20, Tween 80, and PG	HPMC	Atopic Dermatitis (AD)	The NEG-enhanced permeation of oil through skin, as a result of the CM emulgel, augmented AD lesions by reducing oedema, inflammatory cells, and the drying and scaling of skin better than CM alone	[112]
3	Clove oil and Cinnamon oil	Clove and Cinnamon oils	Tween-20	Carboxymethylcellulose	Anti-inflammatory and Anti-nociceptive	A cinnamon NEG had a superior anti-nociceptive effect and inhibitory action on oedema	[89]
4	Clove oil	Glycerol monoacetate (GMA)	Tween 80 and Labrasol	Chitosan, gum acacia, and guar gum	External Inflammation	NEG showed high permeation and prompted enhanced anti-inflammatory action compared to pure clove oil	[113]
5	Curcumin, resveratrol, and thymoquinone	Oleic acid	Tween 20, and PEG 200	Carbopol 940	Psoriasis	NEG formulation incorporated all three active moieties effectively and aided in their permeation through the skin because of the nano size range of NE. Meanwhile, hydrogel improved the drug’s retention on the skin.	[114]
6	Curcumin	emu oil	Cremophor RH 40 and Labrafil M2125CS	Carbopol 940	Rheumatoid Arthritis	Improved anti-inflammatory action was demonstrated by Curcumin NEG due to higher steady state flux (1.71 times), and increased drug retention was shown compared to curcumin dispersion in emu oil and water. Moreover, emu oil ameliorated inflammation via synergistic action.	[115]
7	Curcumin	Myrrh oil	Tween 80 and PG	Sodium CMC	Anti-Inflammatory	NEG formulation enhanced skin permeation of curcumin and exhibited maximum steady-state transdermal flux, showing the maximum enhancement ratio of 7.1; thus, curcumin NEG demonstrated the synergistic action of curcumin and myrrh oil.	[116]
8	Diacerein	Argan oil	Tween 80	Chitosan–Chondroitin Sulfate	Osteoarthritis	NEG demonstrated prolonged drug release with excellent penetration and improved therapeutic activity. The anti-inflammatory properties of chitosan, chondroitin sulfate, and argan oil further enhanced the therapeutic profile of the formulation	[117]
9	Ginger Extract	Isopropyl myristate	Tween 80 and Ethanol	Carbopol 934	Rheumatoid Arthritis	The NEG formulation exhibited good release behavior and spreadability. NEG demonstrated anti-inflammatory action comparable to that of standard ibuprofen.	[118]
10	Mangosteen Rind Extract	Olive oil	PEG 400 and Chromophore RH 40		Anti-inflammatory	NEG formulation enhanced the permeation through skin, and an improved onset of action compared conventional gels. The therapeutic effect was equivalent to diclofenac sodium	[119]
11	*Oldenlandia corymbosa* and *Ageratum conyzoides* Extract	Virgin Coconut Oil	Tween 80 and PEG 400	Carbomer 940	Osteoarthritis (OA)	NEG, whether it is used in combination or as a single extract, has good physical characteristics and shows signs of having a positive impact on cartilage degeneration brought on by MIA through anti-inflammatory action.	[52]
12	*Swietenia macrophylla* oil	*Swietenia macrophylla* oil	sucrose ester	Carbopol 934 and 940	Anti-inflammatory	Anti-inflammatory activity of SM oil was enhanced when applied as a nanoemulgel. This can be attributed to the increased permeation of the nanosized droplets of NEG through a rat skin oil solution	[68]
13	6-Gingerol (GL)	Oleic Acid	Tween 20 and PEG-400	Carbopol 934	Anti-inflammatory	NEG improved skin penetration and significantly enhanced bioavailability of optimized formulation compared to 6-GL-conventional gel, as revealed by dermatokinetic study	[120]

**Table 3:** Nanoemulgels containing synthetic anti-inflammatory drugs.

S.No.	Active Moiety	Oil	Surfactant and Co-Surfactant	Gelling Agent	Disease	Inference	Reference
1	Aceclofenac	Oleic acid	Tween 20, ethanol	Carbopol-940	Arthritis	The NEG showed a higher drug release and permeation rate in vitro and ex vivo than the simple gel, resulting in a significant enhancement in bioavailability	[124]
2	Aceclofenac	Labrafil and Triacetin	Cremophor EL and tween 80 and Transcutol HP and PEG 400	Carbopol-940	Osteoarthritis (OA)	NEG showed a twofold flux compared to that of the marketed Aceclofenac sample. Overall, it shows a higher permeation rate and permeation coefficient.	[125]
3	Betamethasone Dipropionate	Babchi oil and eucalyptus oil	Tween 20 and ethanol		Psoriasis	NEG formulation-enhanced drug permeation sustained its release and showed good spreading properties. The formulation demonstrated a better inhibition of oedema than that of marketed formulation	[126]
4	Celecoxib	Acetonitrile, triacetin, campul 908P	Acconon MC8-2EP and Capmul MCM C-10	Carbopol-940	Rheumatoid arthritis	The NEG formulation exhibited a good drug release profile, enhanced ex vivo permeation (3 times more than that of conventional gel), and thus higher anti-inflammatory activity.	[127]
5	Desonide	Eucalyptus oil, Oleic acid and Triacetin	Tween 80, Span 80 and Poloxamer 407	Carbopol 980	Anti-Inflammatory	NEG improved drug permeation through skin, and prolonged drug release, resulting in improved dosing frequency	[128]
6	Diclofenac sodium	cumin essential oil	Tween 80, Tween 20, or a mixture of Tween 80/Span 80	Carbopol 940	Inflammatory pain	NEG improved the permeation by 1.5 times and demonstrated better antinociceptive activity compared to the simple gel and marketed preparation	[129]
7	Diclofenac sodium	Clove oil, isopropyl myristate, eucalyptus oil and peppermint oil	Tween 20 and PEG 400	Carbopol 980	Analgesic and Anti-Inflammatory	Diclofenac sodium-loaded emulgel showed better in vitro drug release, and enhanced analgesic and anti-inflammatory activity compared to simple and marketed Diclofenac sodium gel	[130]
8	Diflunisal (DIF) and solubility enhanced diflunisal (DIF-IC)	Eucalyptus oil	Tween 80 and Transcutol-P	Sodium alginate, carboxymethylcellulose sodium and xanthan gum	Rheumatoid arthritis	DIF-IC-laden NEG showed better in vitro skin permeation than simple DIF NEG. DIF-IC NEG prepared with XG demonstrated superior anti-inflammatory activity in vivo	[131]
9	Etoricoxib (ETB)	Eucalyptus Oil	Tween 20, and PEG 200	carbopol 934	Inflammation and pain	ETB-loaded NEG increased the permeability coefficient by more than 1.5-fold and improved the analgesic and anti-inflammatory effect in vivo	[132]
10	Flurbiprofen	Camphor methyl salicylate, linseed oil and triacetin	tween 80, propylene glycol	Carbopol 934	anti–inflammatory	The optimized NEG significantly enhanced the permeability and exhibited a substantial surge in the inhibition of inflammation compared to the marketed gel	[133]
11	Ketoprofen	Oleic acid	Tween 80, and Transcutol P	Carbopol 940	anti-inflammatory (rheumatism)	NEG showed better retention, and high cumulative drug penetration and flux with low lag duration compaerd to the marketed product	[134]
12	Lornoxicam	Labrafac	Tween 80 and Pluronic F68	Carbopol 934	Anti-inflammatory	NEG showed almost 3 times more in vitro drug release than the conventional gel. The permeability, steady-state flux, coefficient, and enhancement ratio were significantly higher for the nanoemulsion based gel than the simple gel	[135]
13	Meloxicam	Eucalyptus Oil	Tween 80 and PEG 400	HPMC	Anti-inflammatory	NEG showed high drug permeation and enhanced anti-inflammatory action due to the synergistic effect of meloxicam with eucalyptus oil	[136]
14	Meloxicam	Caprylic acid	Tween 80 and the Propylene glycol	Carbopol 940	Anti-inflammatory	NEG increased the permeation and penetration by reducing the subcutaneous barrier and showed exceptional efficacy in managing inflammation compared to the drug solution	[137]
15	Piroxicam	Oleic acid	Tween 80 and ethanol	Carbopol 934	Anti-inflammatory	NEG exhibited higher drug permeation and flux. Moreover, it showed better drug retention and a reduced lag time compared to the marketed preparation	[138]
16	Tofacitinib (TFB)	Oleic acid	Tween 80 and propylene glycol	Carbopol-934	Rheumatic arthritis	NEG formulation showed higher drug-loading efficiency and enhanced permeation via skin	[139]

**Table 4:** Recent patents on nanoemulgels, specific to inflammatory conditions.

Application No.	API	Title	Indication	Inventors	Year of Publication/Grant	Reference
CA3050535C	Nutraceuticals possessing anti-inflammatory properties such as resveratrol, polyphenols, cinnamaldehyde, lipoic acid, etc.)	Methods of treating inflammatory disorders and global inflammation with compositions comprising phospholipid nanoparticle encapsulations of anti-inflammatory nutraceuticals	Inflammatory disorders	Nanosphere Health Sciences Inc.	2019	[145]
202021044492	*Ginger oleoresin* and lipid guggul extract	Topical NEG formulation for arthritic inflammation and pain	Arthritic Inflammation and Pain	Dr. Munira Momin	2022	[146]
202221026634	*Clerodendrum inerme* ethanolic extract	Novel nanoemulgel formulation containing extract of *Clerodendrum inerme* for management of psoriasis	Psoriasis	Gaikwad Ravindra Ganpati	2022	[147]
202111020026	Curcumin	NEG formulation for topical delivery of curcumin	Anti-inflammatory, antioxidant, angiogenesis and anti-proliferative	Kiran Singh Sharma, Dr. Jagannath Sahoo	2021	[148]
